# Gene Activation through the Modulation of Nucleoid Structures by a Horizontally Transferred Regulator, Pch, in Enterohemorrhagic *Escherichia coli*

**DOI:** 10.1371/journal.pone.0149718

**Published:** 2016-02-22

**Authors:** Naoki Fukui, Taku Oshima, Takeshi Ueda, Naotake Ogasawara, Toru Tobe

**Affiliations:** 1 Department of Biomedical Informatics, Graduate School of Medicine Osaka University, 1-7 Yamadaoka, Suita, Osaka, 565-0871, Japan; 2 Graduate School of Biological Science, Nara Institute of Science and Technology, 8916-5 Takayama, Ikoma, Nara, 630-0101, Japan; Centre National de la Recherche Scientifique, Aix-Marseille Université, FRANCE

## Abstract

The horizontally transferred chromosomal segments, which are the main source of genetic diversity among bacterial pathogens, are bound by the nucleoid protein H-NS, resulting in the formation of a nucleoprotein complex and the silencing of gene expression. The de-silencing or activation of virulence genes necessary for the colonization of enterohemorrhagic *Escherichia coli* is achieved mainly by the action of two regulators, Pch and Ler, which are encoded by horizontally transferred elements. Although Ler has been shown to activate transcription by counteracting H-NS silencing, the mechanism for Pch is poorly understood. We show here that Pch activates the *LEE1* promoter and also enhances the Ler-mediated activation of other LEE promoters. Transcriptional activation was completely dependent on repression by the H-NS/StpA/Hha/YdgT complex, indicating that Pch-derived activation was achieved by alleviating H-NS-mediated silencing. Expression of *pch* reduced the binding of H-NS at *LEE1* promoter and altered the nucleoprotein complex. Furthermore, *in vitro* reconstruction of the protein-DNA complex on *LEE1* promoter DNA confirmed the exclusive effect of Pch on H-NS binding. These results demonstrated that Pch is another anti-silencing regulator and a modulator of H-NS-containing nucleoprotein complexes. Thus, the anti-silencing mechanism plays a key role in the coordinated regulation of virulence genes in EHEC.

## Introduction

The acquisition of external genetic information by bacteria is one of the dynamic forces underlying the emergence of genetic diversity, allowing an organism to adapt to a novel environment. The sequencing of a variety of bacterial genomes has revealed strain- and subspecies-specific genomes as well as the common background genome that characterizes a species. The genome of pathogenic bacteria is often distinguished from that of non-pathogenic strains of the same species by the presence of virulence genes on pathogen-specific genomic islands. Such genomic segments commonly reside in mobile genetic elements, such as plasmids, phages, conjugative transposons, and integrons [1]. The horizontal transfer of genes is believed to be the main mechanism by which bacteria acquire new virulence traits [2, 3]. The combination and cooperation of many virulence traits in different elements scattered around the chromosome and in plasmids determine the characteristics and infectious behavior of pathogens. The acquisition of similar genetic elements often causes the parallel evolution of strains of the same pathotype from separate phylogenetic groups [4].

While the incorporation of exogenous genes is important for bacteria to acquire new abilities to adapt and survive in changing environments, the controlled expression of these exogenous genes is essential for maintaining the organism’s physiological activity. In *Salmonella* Typhimurium and *Escherichia coli*, it has been shown that the silencing of laterally acquired genes is mediated by selective binding at AT-rich regions of the nucleoid protein H-NS [5–7]. Because many virulence traits in these species come from horizontally acquired genes and the sequences of these genes are AT-rich, H-NS has been identified as a negative regulator of many virulence genes. Moreover, a deficiency in H-NS sometimes causes the upregulation of virulence genes. To date, H-NS and its related nucleoid-associated proteins have been shown to play important roles in the regulation of virulence genes in a variety of enteric pathogens [8–10].

Enterohemorrhagic *E*. *coli* (EHEC) is a human pathogen that causes a broad spectrum of illnesses, ranging from mild diarrhea to severe diseases, such as hemorrhagic colitis and hemolytic uremic syndrome. EHEC belongs to a group of A/E pathogens that are defined by their ability to form a histopathological feature known as attaching and effacing (A/E) lesions [11]. A/E lesions are characterized by the localized destruction of brush border microvilli and the intimate attachment of bacteria to the membrane of host cells [12]. The genes essential for causing the A/E lesions are encoded in a pathogenicity island called the locus of enterocyte effacement (LEE). The LEE consists of more than 40 genes that encode a type III secretion system (T3SS), secreting proteins, chaperones, adhesin, and transcription regulators [13]. The transcription of LEE genes is positively regulated by Ler, which is also encoded by LEE, and by Pch regulators, which are encoded at other loci and activate *LEE1* transcription, including the *ler* gene. The expression of many genes at different loci on the chromosome are controlled in coordination with the LEE genes, through the action of Pch and Ler [14].

The Pch/Ler-activated genes are divided into two classes, L1 and L2, according to their dependency on Ler for their transcriptional activation: the transcriptional activation of genes in class L1 requires Ler and Pch, while those in class L2 are less dependent on Ler and can be activated by Pch alone[14]. Ler is a small DNA-binding protein that shares homology with H-NS in its DNA-binding domain [15]. Pch is a small transcriptional regulator that lacks homology with known DNA-binding proteins [14, 16]. Both Ler and Pch are encoded on genetic mobile elements[17] and are likely to be acquired through horizontal transfer, similar to virulence genes [17]. Pch and Ler bind to many sites on the chromosome, including in the *E*. *coli* K12 common backbone regions. The binding sites on virulence genes are found at the coding region as well as at downstream and upstream sequences. Ler has been shown to activate transcription through its anti-H-NS activity [15, 18–22], except for the *LEE1* promoter, on which Ler functions as an autorepressor by forming a DNA loop at the promoter region [23]. On the other hand, although Pch and its homolog in enteropathogenic *E*. *coli* (EPEC), PerC, have been shown to activate the *LEE1* promoter [14, 16, 24, 25] and suggested to be an anti-repressor for H-NS-silencing as well as an activator [25], the molecular mechanism for this activation remains unsolved. Contribution of Pch or PerC regulator in the activation of the *LEE1* promoter seems different between EHEC and EPEC, since transcription of LEE genes in EPEC is dependent on PerC only under the specific conditions, such as static in the presence of CO_2_ [25], while the transcription of LEE genes in EHEC is dependent on Pch even under shaking condition [14, 16, 26]. Furthermore, the *pch* genes are essential for the expression of LEE genes and EHEC virulence [16], in contrast to the *perC* gene of EPEC, in which LEE expression is unaffected by the absence of the *perC* gene[27]. In addition, *LEE1* promoter of EHEC is activated in *E*. *coli* K-12 background only in the presence of *pch* gene [28], while the cloned LEE genes of EPEC is expressed in *E*. *coli* K-12 in the absence of PerC [29].

In this study, we explored the role of Pch in LEE expression and the effects on H-NS-containing nucleoprotein complex formation at the *LEE1* promoter. The *LEE1* promoter was activated by Pch only when the promoter activity was repressed by H-NS and other related proteins. LEE and other virulence loci of EHEC were bound by H-NS/StpA, but the occupancy of H-NS/StpA was reduced by the expression of *pch* in addition to *ler*. Furthermore, the binding of H-NS to *LEE1* promoter DNA was shown to be inhibited by Pch. These findings supported a mechanism in which the activation of gene expression was achieved by reducing H-NS/StpA binding by competitive binding of Pch.

## Results

### Pch is required for full activation of LEE genes and adherence capacity

**Fig 1:**
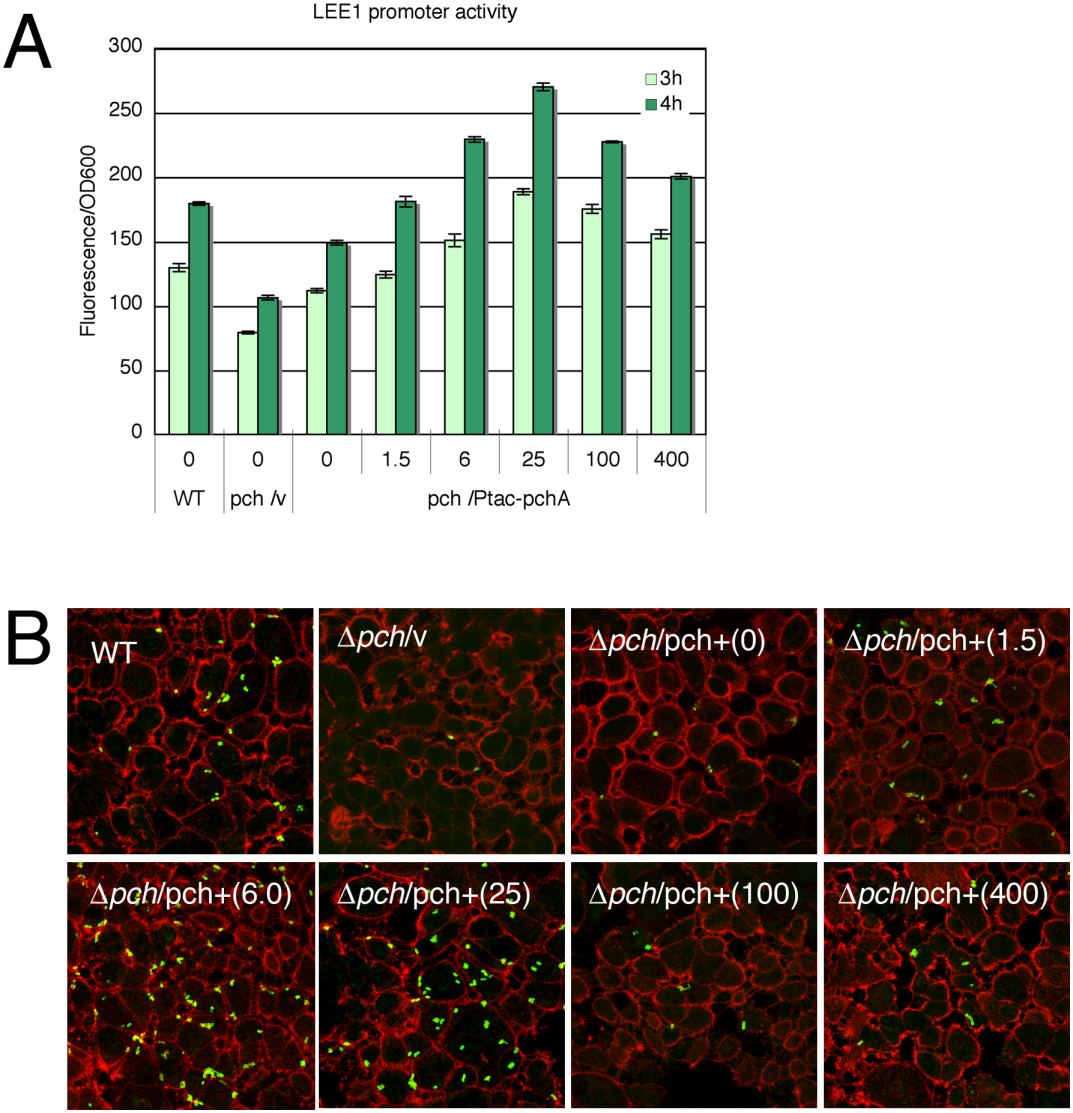
Activation of LEE gene expression and adherence capacity by increasing *pch* expression. A. Dose effect of Pch on *LEE1* promoter activity. The *pch* mutant of EHEC harboring the P*tac-pchA* operon fusion plasmid (pTB101-*pchA*) and P*LEE1*-gfp operon fusion plasmid (pSU-gfp-P*LEE1*) was grown in DMEM containing various amounts of IPTG. *LEE1* promoter activity was measured as expression levels of GFP, which was controlled by the *LEE1* promoter. The activities were assayed at 3 or 4 h from the start of bacteria growth after a 100-fold dilution of overnight culture. B. Dose effect of Pch on adherence capacity. After growth in DMEM containing various amounts of IPTG (shown in parenthesis), bacteria were incubated with Caco-2 cells, and adherent bacteria were visualized by fluorescent staining with an anti-O157 LPS antibody and an Alexa488 secondary antibody. F-actin was visualized by staining with rhodamine-phalloidine.

Transcription of the *LEE1* operon, which includes the *ler* gene, was positively regulated by Pch, which is encoded on other prophage-like elements. Although growth in DMEM enhanced the expression of LEE genes, compared with growth in LB, the level of LEE gene expression in DMEM nevertheless could have been lower than its level when fully activated. Because the optimal conditions for the full expression of LEE genes and other LEE-associated genes are currently unknown, we attempted to achieve the full expression of these genes artificially by inducing a high level of the positive regulator PchA, which also increases *ler* expression, in wild type of EHEC O157 Sakai. Transcription of the *pchA* gene was increased by placing the gene downstream of an IPTG-inducible *tac* promoter and by adding IPTG to the growth medium. The promoter activity increased with increasing IPTG concentrations of up to 25 μM (Fig 1A). High concentrations of IPTG, e.g., 100 and 400 μM, decreased the *LEE1* promoter activity because IPTG was detrimental to the growth of the bacteria harboring P*tac*-*pchA* plasmid at these concentrations (S1 Fig). As expected, the promoter activity reached much a higher level than that in the wild-type strain by inducing *pchA* expression. Because the *LEE1* operon encodes a regulatory protein, Ler, for other *LEE* genes and operons, activation of the *LEE1* promoter enhanced the expression of whole *LEE* genes, which contributed to adherence capacity. To explore the effect of *pchA* induction on adherence capacity, Caco-2 cells were infected with EHEC-harboring P*tac*-*pchA* after grown in DMEM containing various concentration of IPTG. In accordance with the *LEE1* promoter activity, the efficiency of adherence increased with increasing IPTG concentrations of up to 25 μM, and the efficiency reached much a higher level than that in the wild type (Fig 1B). These results indicated that the expression of *LEE* genes and, hence, the expression of adherence capacity could be achieved at a much higher level by enhancing *pch* expression.

**Fig 2:**
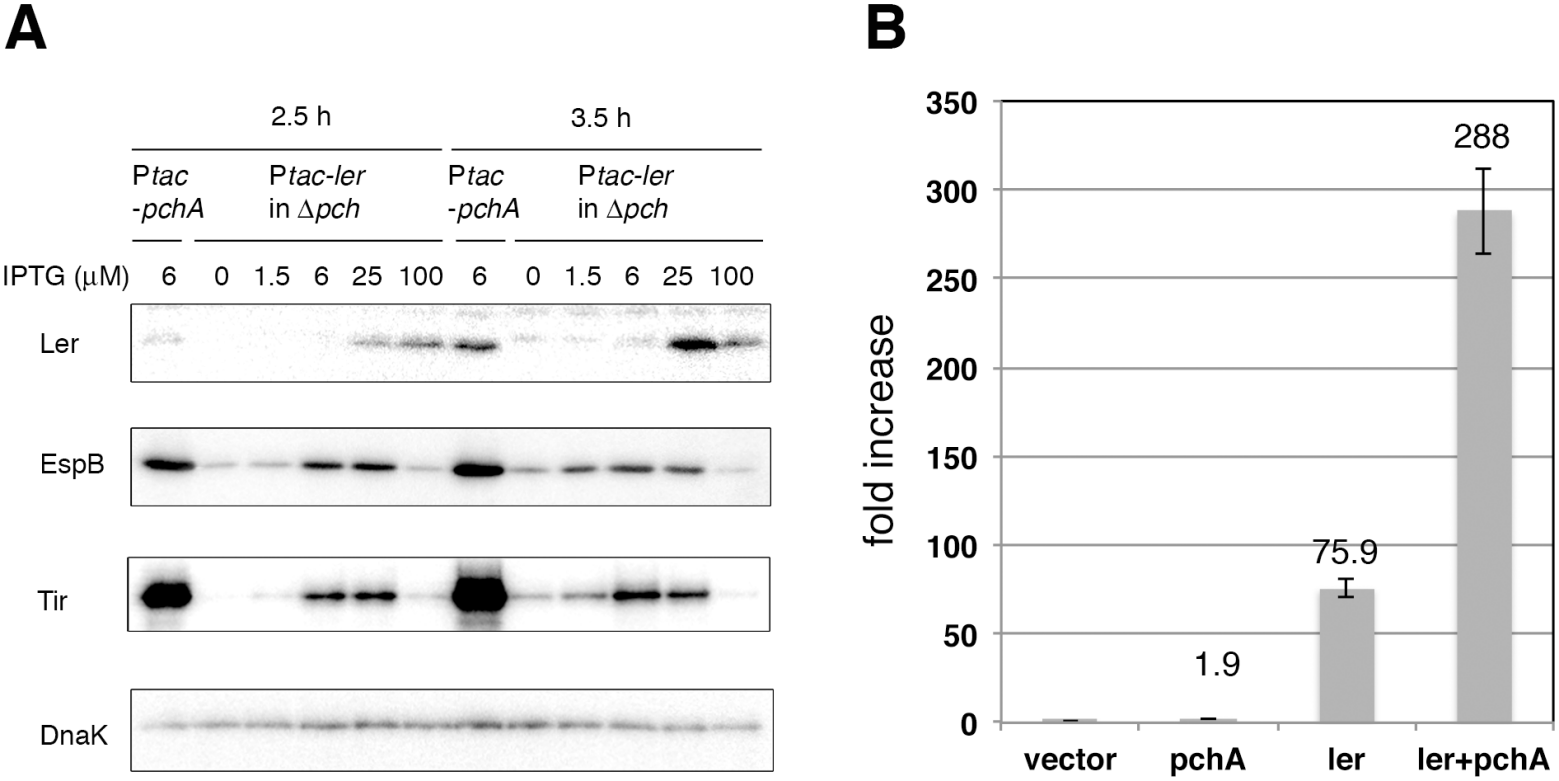
Activation of LEE genes in the presence or absence of Pch. A. Dose effect of Ler on LEE gene expression. The EHEC *pch* mutant (Δ*pchA*Δ*pchB*Δ*pchC*) harboring the P*tac-ler* operon fusion plasmid pTB101-*ler* was grown in DMEM containing various amounts of IPTG for 2.5 or 3.5 h, after starting its growth in fresh medium. The production of Ler, EspB and Tir was monitored by immunoblotting. As a reference, the production of these proteins in the *pch* mutant harboring P*tac-pchA* plasmid pTB101-*pchA*, grown in DMEM containing 6 μM IPTG, is shown. The DnaK level was the quantitative control. B. Activation of *LEE2* promoter activity by Pch and Ler. W3110 harboring pLux-P*LEE2* and pTB101 (vector) or P*tac-pchA* plasmid pTB101-*pchA* (pchA) or P*tac-ler* plasmid pTB101-*ler* (ler) or P*tac-ler* plasmid pTB101-*ler* and P*lac-pchA* plasmid pSU-*pchA* (ler+pchA) were grown in LB containing 6 μM IPTG at 37°. Luciferase activity was measured when the OD_600_ reached approximately 1.0, and the luciferase value was normalized according to the OD_600_. The activity is presented as a fold increase compared to the activity of the vector control.

The role of Pch in the expression of the LEE genes was then examined by comparing the efficiency of gene activation by Ler with or without Pch. When Ler was produced from the P*tac-ler* fusion gene, the production of EspB, which is encoded by the *LEE4* operon, and Tir, encoded by the *LEE5* operon, increased in parallel with the increase in Ler, even without Pch. However, the levels of the EspB and Tir proteins remained much lower than those in the strain expressing the same amount of Ler along with Pch (Fig 2A). This result clearly indicated that Pch contributed to the full expression of the LEE genes, although Ler alone could activate some expression.

To explore the positive effect of Pch on LEE gene transcription, we measured the promoter activity of the *LEE2* operon in *E*. *coli* K-12 strain W3110 with or without *pch* genes (Fig 2B). The *LEE2* promoter was at a very low level (basal level) without Ler and Pch, while the activity increased in the presence of Ler to 76-fold that of the basal level. In contrast, Pch alone showed a slightly positive effect on the promoter activity (1.9-fold increase from the basal level). In the presence of both Ler and Pch, the promoter activity was enhanced and reached a much higher level than Ler alone (288-fold of the basal level and 3.8-fold of Ler alone). The results confirmed that full activation of the *LEE* promoters by Ler occurred in cooperation with Pch.

### Pch achieves promoter activation by de-repression of H-NS/StpA/Hna/YdgT-mediated silencing

**Fig 3:**
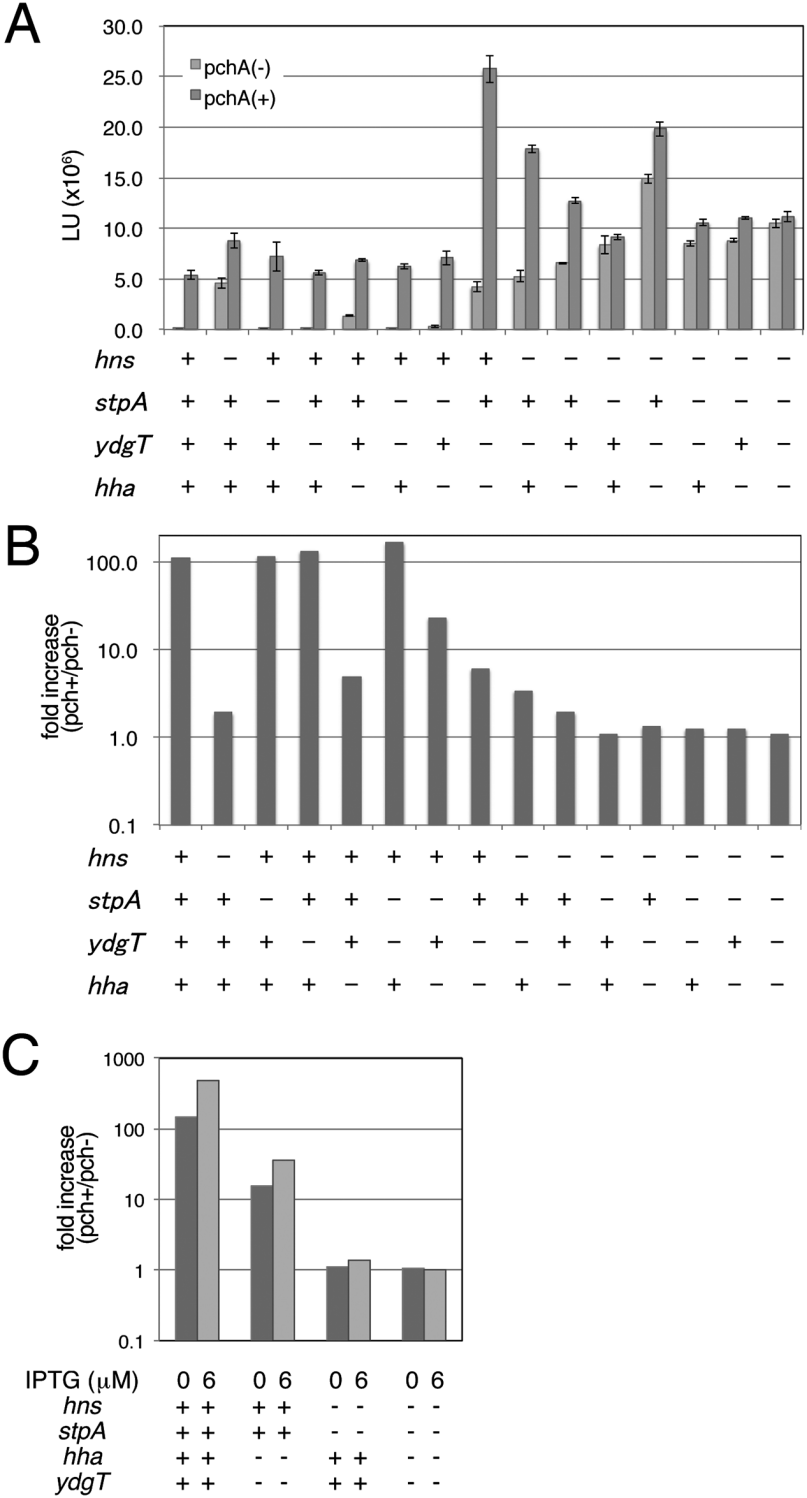
Activation of the *LEE1* promoter by Pch in the presence or absence of H-NS and related proteins. A. Effect of Pch on *LEE1* promoter activity in deletion mutants of *hns* and its homolog and paralog genes. W3110 and its derivative mutants harboring pLux-P*LEE1* and pTB101 (-) or pTB101-*pchA* (+) were grown in LB. Luciferase activity was measured when the OD_600_ reached approximately 2.0, and the luciferase value was normalized by the OD_600_. B. Activation by Pch in the mutants. The activation rate in each strain was deduced from the experiments in A. C. Loss of dose-dependent activation. Bacteria were grown in LB containing 0 or 6 μM IPTG, and luciferase activity was measured when the OD_600_ reached approximately 2.0. Relative activity against the activity in the same strain without *pch* is shown as a fold increase. As shown in the WT strain (possesses all four genes), even without IPTG, *pchA* was expressed from P*tac*-*pchA* and activated the *LEE1* promoter.

Because *E*. *coli* possesses an additional H-NS paralog, StpA, and H-NS homologs, Hha and YdgT, and the involvement of these proteins in gene silencing of subset of genes has been suggested[30], we explored the roles of these proteins as well. Although Hha does not have a DNA-binding domain, Hha is believed to repress gene expression by modulating H-NS-containing nucleoprotein complexes by interacting with H-NS [30, 31]. It has been shown that the *LEE1* promoter of EHEC is activated by Pch without Ler and is negatively regulated by H-NS and Hha [14, 18, 32]. To clarify the mechanism of promoter activation by Pch, the activity of the *LEE1* promoter in the presence or absence of the *pch* gene was compared in a series of deletion mutants for genes encoding H-NS and its paralogs and homologs. Since *hns* gene seems to be essential for growth of EHEC, we choose to make a set of mutations in *E*. *coli* K-12 strain W3110. A plasmid, pTB101-*pchA* or pTB101 (for vector control), was introduced into W3110, which does not possess *ler* gene, and its derivatives harboring pLux-P*LEE1*, and luciferase activity was measured. In the W3110 wild type (all four genes, *hns*, *stpA*, *hha* and *ydgT*, are present), the promoter activity remained at a low level without Pch, although the activity was enhanced 110-fold in the presence of Pch even at a basal level expression without IPTG (Fig 3A and 3B). Then, we measured *LEE1* promoter activity in derivatives of W3110 that possess various combinations of deletion mutations of *hns*/*stpA*/*hha*/*ydgT*, with or without *pch*. Since some combination of mutation affected the growth, the promoter activity was measure at the same growth phase such as early stationary phase (S2 Fig). Deletion of the *hns* gene resulted in an increase in the promoter activity even without *pch*, and it reduced the positive effect of Pch to only 1.9-fold. Although the deletion of the *stpA* gene alone did not affect the promoter activity or positive effect of Pch, simultaneous deletion of *hns* and *stpA* diminished the effect of Pch completely. The results indicated that StpA functioned as a backup of H-NS only in part and that repression by H-NS and StpA was necessary for activation by Pch. Hha is believed to be a part of an H-NS-containing nucleoprotein complex. Deletion of the *hha* gene increased the promoter activity even without Pch, resulting in a decrease in Pch-mediated activation to 5.0-fold. This negative effect was completely abrogated by additional deletion of *hns* and *stpA*. In contrast to Hha, deletion of *ydgT*, encoding an Hha homolog, did not affect the Pch-mediated activation even in the absence of Hha though basal promoter activity was affected. These results indicated that Hha contributed to the repression of the *LEE1* promoter in part and that its effect was dependent on H-NS and StpA. Complete activation of *LEE1* promoter by Pch was observed only when the repression nucleoprotein complex consisted of H-NS/StpA and Hha. To further examine the dependence of Pch on H-NS and related proteins for the activation of the *LEE1* promoter, expression levels of *pch* were more enhanced by adding IPTG to the medium (Fig 3C). In the wild type, the promoter activity was enhanced and reached a higher level than the activity in the strain harboring P*tac-pchA* grown without IPTG. In the *hha ydgT* mutant, although the magnitude of enhancement was decreased, induction of *pch* by adding IPTG increased the activity. In contrast, no effect of the induction of *pchA* was shown in the strain with deletion of the *hns* and *stpA* genes. The results supported the necessity of repression by the H-NS-containing nucleoprotein complex for the activation of the *LEE1* promoter by Pch, and the de-repression activity of Pch was independent on Ler.

### Distinct bindings of Ler and Pch from H-NS/StpA-binding sites

**Fig 4:**
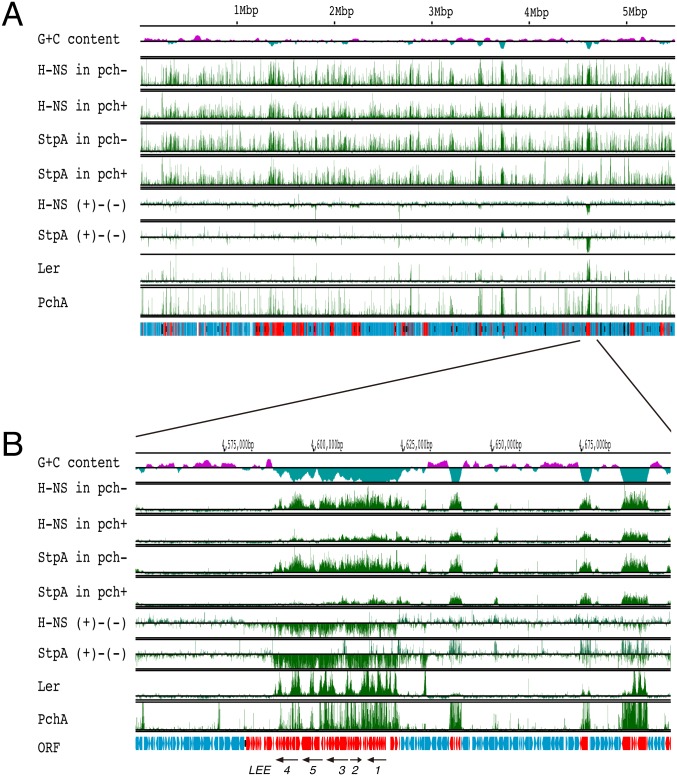
Effects of *pch* expression on H-NS and StpA bindings. A. Distribution of H-NS and StpA binding over the EHEC O157 Sakai chromosome. The top column shows G+C content. 2^nd^ and 3^rd^ graphs show H-NS binding in EHEC deficient in *pch* (pch-) and a *pch*-expressing strain (pch+). The 4^th^ and 5^th^ graphs show StpA binding in EHEC deficient in *pch* (pch-) and a *pch*-expressing strain (pch+). The 6^th^ and 7^th^ columns show differences in H-NS and StpA binding between pch (-) and pch (+) strains. The 8^th^ and 9^th^ columns show Ler and Pch binding from previous data [14], respectively. The bottom column shows ORFs in the *E*. *coli* K12-common chromosome (blue) and ORFs in the LEE and in the other laterally transferred elements (red). B. Distribution of H-NS binding in the LEE and surrounding region of EHEC O157 Sakai. Arrows at the bottom show the positions of the *LEE* operons. The resolution was estimated around 50bp from density of microarray and fragmentation of DNA.

The genes in the LEE and the effector genes outside of the LEE were positively regulated by two DNA-binding proteins: Pch and Ler [14]. In our previous study, we determined the binding sites for Pch and Ler on the EHEC O157 Sakai chromosome [14]. The binding sites for both proteins were significantly low in G+C content on the chromosomal regions of the *E*. *coli* backbone as well as in mobile elements. To compare the binding preferences of Pch and Ler with those for H-NS and StpA, we examined the binding of H-NS or StpA to the EHEC chromosome, using the ChIP-chip method. To avoid the effects of virulence activators, the H-NS/StpA binding profile was first determined in the EHEC *pch* mutant strain, which does not express Pch and Ler (Fig 4, second and fourth column), harboring FLAG-tagged *hns* gene or FLAG-tagged *stpA* gene. We isolated approximately 440 loci with H-NS binding on the chromosome, and the binding profile of StpA was almost identical to that of H-NS. The G+C content of the binding sites was lower than the average for the whole *E*. *coli* chromosome in most cases, as expected (Fig 4). Among these sites, 189 loci were found on chromosomal segments unique to EHEC O157 Sakai, which were mostly phage or phage-like segments, compared to *E*. *coli* K-12 (Fig 4). All of the known virulence genes on phage-like elements were closely associated with H-NS binding, as expected, although the size and location of the binding site varied according to the gene. In particular, the entire 35-kb LEE region was covered with H-NS (Fig 4B). In addition, H-NS binding was found on prophage-like segments on all of the effector genes that are known to be expressed coordinately with LEE genes (S3 and S4 Figs).

**Fig 5:**
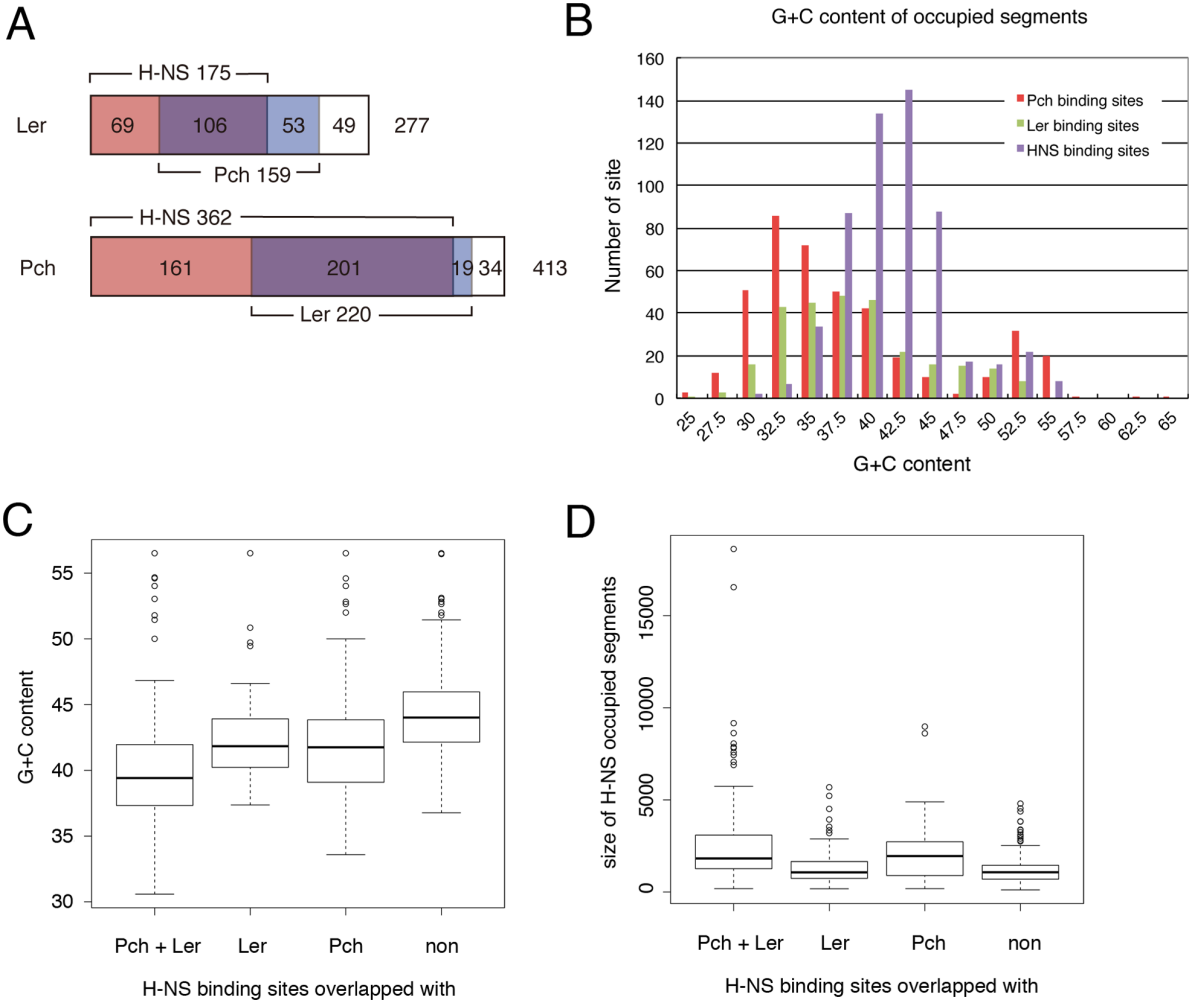
Features of binding sites for Pch and Ler. A. Overlapping of binding sites. Binding sites for Ler and Pch are classified by overlapping with H-NS bindings and Pch or Ler bindings. B. G+C contents of binding sites for H-NS, Pch, and Ler. Distributions of a number of binding sites with various ranges of G+C contents are shown. For example, 25 indicates that the G+C content of the binding site sequence is less than 25%. C. Distributions of the G+C content of the binding site for H-NS with or without Pch- and/or Ler-binding sites. Box plots represent medians (horizontal lines), the upper and lower quartile values (boxes), and the most extreme data points within 1.5 interquartile ranges (whiskers). D. Distributions of the sizes of the binding sites for H-NS with or without Pch- and/or Ler-binding sites.

Comparison of the Pch and Ler binding sites, which were previously published [14], with those of H-NS showed that although most of them overlapped, there were clear differences between them (Fig 4). Eighty-eight percent of the Pch-binding regions and 63% of the Ler-binding regions were at binding regions for H-NS (Fig 5A). The H-NS-binding regions were distinct from the regions where Pch and/or Ler bound in many cases, with only 32% of the binding regions for H-NS being common to Pch- and/or Ler-binding regions. Different features were found between H-NS-binding regions and Pch- and/or Ler-binding regions. The size of the DNA fragment associated with the binding of Pch or Ler was smaller than that associated with H-NS: the average sizes for Pch-binding sites and Ler-binding regions were 453 bp and 1105 bp, respectively, while that of the H-NS-binding regions was 1643 bp. Furthermore, the G+C content of the Pch- or Ler-binding regions was lower than that of the H-NS-associated regions, and that of the Pch-binding regions was lowest among them (Fig 5B). Even for the H-NS binding regions, the G+C content of the region containing Pch- and/or Ler-binding regions was lower than that of the regions without them, and the size of the regions containing Pch- and/or Ler-binding regions was larger than that of the regions without them (Fig 5C and 5D). Interestingly, the G+C content was much lower, and the size was much larger for the H-NS binding regions containing both Pch- and Ler-binding regions than for the regions with only one of them. Furthermore, this tendency was obviously shown when comparing H-NS binding regions with altered H-NS binding by PchA/Ler expression and those with unaltered bindings (S5 Fig). These results indicated that the preferential sequence of Pch binding was different from that of H-NS binding but that most of the Pch-binding regions were located in H-NS binding regions. Because the DNA-H-NS complex has been shown to form by the initial binding of H-NS to core DNA-binding sites, followed by extension of the binding to flanking regions [33], and the sequence of the H-NS-binding regions containing Pch- and Ler-binding regions is relatively AT-rich and larger in size, it is likely that the binding regions for Pch and Ler would be located in the extended binding regions beside the core binding regions for H-NS.

### Alteration of H-NS/StpA binding by the activation of Pch and Ler expression

To explore the effects of Pch and Ler production on H-NS binding, we first compared the H-NS-binding profiles of wild-type EHEC and a *pch* (Δ*pchA* Δ*pchB* Δ*pchC*) mutant of EHEC, grown in DMEM. However, little to no obvious changes in the H-NS-binding profiles were observed (data not shown). Then, we used medium containing 6 μM IPTG to determine the effects of Pch and Ler on the binding of H-NS/StpA to the chromosome of EHEC *pch* mutant harboring P*tac-pchA*, which is hereafter called the Pch+ strain. Induction of *pchA* expression resulted in simultaneous enhancement of production of Ler as well as PchA in EHEC.

The relative intensities and patterns of the DNA bound by H-NS in the strain expressing PchA and Ler were the same as in EHEC without Pch and Ler for most of the H-NS-binding sites (Fig 4). However, at the regions encoding virulence genes, the H-NS-binding profiles were altered when both PchA and Ler were expressed (Fig 4A, columns, H-NS (+)-(-) and StpA (+)-(-)). In particular, the relative amount of DNA bound to H-NS decreased dramatically over the entire LEE sequence by the expression of PchA and Ler (Fig 4B, Pch+), compared with the binding levels in EHEC deficient in *pch* and *ler* expression (Pch-). Decreased binding of H-NS was also observed at regions containing non-LEE effector genes belonging to both regulatory classes L1 and L2 [14], such as the *espJ-tccP*, *nleA-nleM1* (S3 Fig), *espX7-espN*, and *nleC-nleH1-2-nleD* regions and the *nleG* clusters (S4 Fig). In most cases, the H-NS binding was not completely abolished by the expression of PchA and Ler; rather, the relative intensities were decreased, and the binding patterns were altered. The binding of PchA and Ler was closely correlated with these changes; that is, the sites with an altered binding pattern were bound by PchA and Ler (Fig 4A). These results suggested that the binding of PchA and Ler at least reduced the occupancy of H-NS at DNA regions corresponding to virulence-associated genes.

To distinguish the role of PchA from that of Ler in altering H-NS-binding, the H-NS binding profile in EHEC expressing only Ler was determined. The H-NS binding profile in this strain (called the Pch-Ler+ strain) was almost the same as in the EHEC strain lacking both regulators (S6 Fig, second circle). Although the LEE genes, except for those in the *LEE1* operon, were greatly activated by Ler without Pch, as shown in Fig 2, the H-NS binding to these regions was barely affected by the expression of Ler alone (S5 Fig, H-NS in Pch-Ler+). H-NS binding to other regions for effector genes was also unaltered by the induction of Ler expression in the absence of Pch production (S5 Fig, H-NS in Pch-Ler+). In contrast, the expression of both PchA and Ler drastically reduced H-NS binding to these regions. These results were consistent with the observation that Pch contributed to the full expression of the LEE genes, although Ler alone could activate some expression.

### Pch reduces H-NS/StpA binding at the *LEE1* promoter region

The reduction in H-NS-bound DNA in EHEC expressing PchA and Ler at Pch/Ler-binding sites suggested that an alteration in the H-NS-DNA complex occurred as a result of the binding of both PchA and Ler. In addition, the expression of *pchA* resulted in the activation of the *LEE1* promoter without Ler (shown in Fig 3), suggesting that PchA alone could affect the H-NS-DNA complex. Although a reduction in and alteration of H-NS-binding at the *LEE1* promoter region were shown, H-NS binding was still observed, even in the strain expressing PchA. Because the ChIP-chip technique could not distinguish long DNA fragments in a large complex from short DNA fragments in a small complex, we could not determine whether the reduced signals were caused by a decrease in the total binding of H-NS or by changes of large complexes into small complexes. Therefore, to distinguish large complexes from small complexes, we performed PCR for different-sized segments representing a given region, after isolating H-NS-bound DNA using the ChIP method.

**Fig 6:**
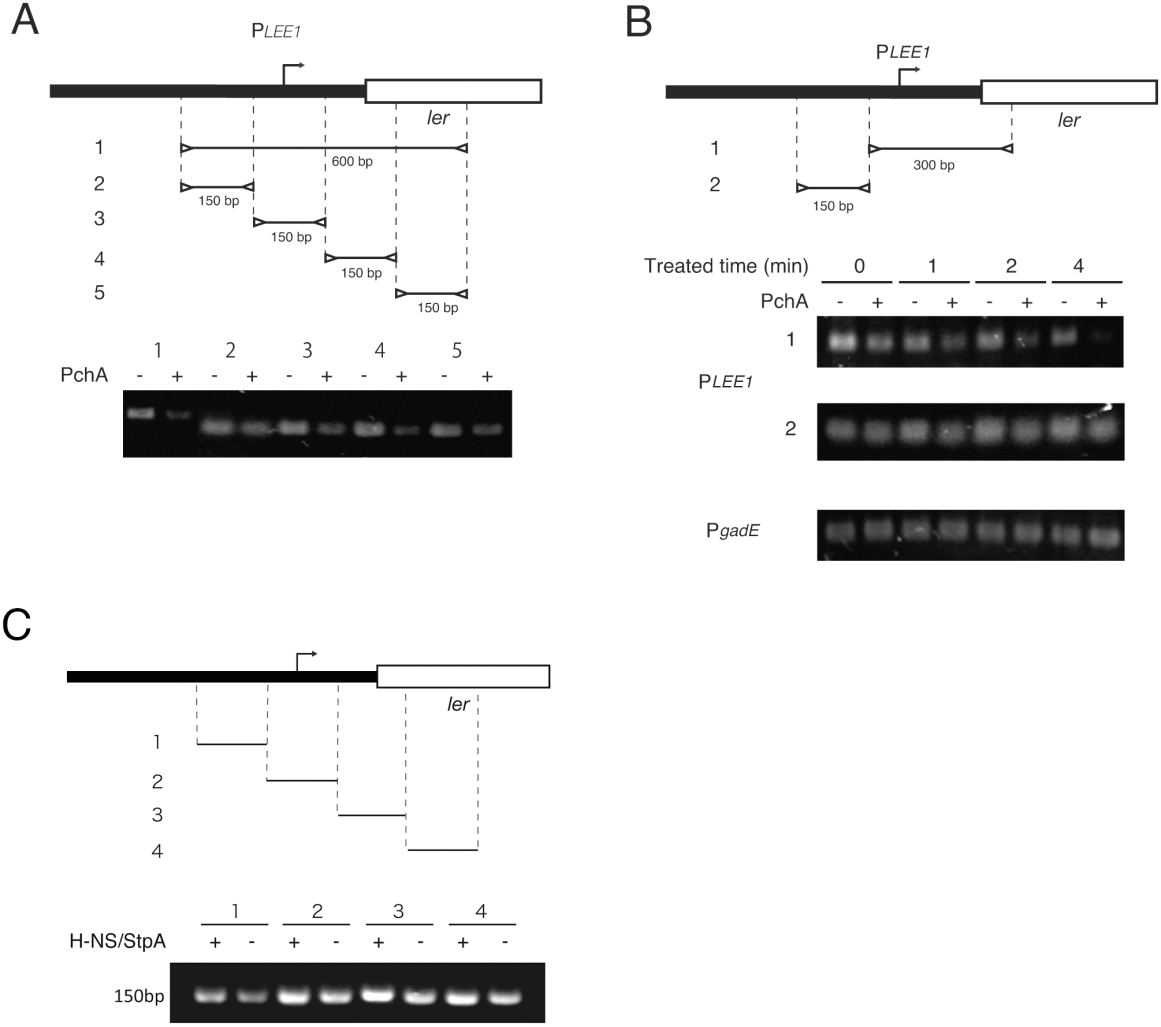
Changes in H-NS-containing nucleoprotein complexes by *pchA* expression. A. Difference in the effect of PchA on H-NS binding at the *LEE1* promoter region. ChIP-purified DNA from EHEC expressing PchA (Pch+) or deficient in *pch* (Pch-) was used as templates in PCR for various segments (1 to 5). B. Difference in sensitivity to hydroxyl radical attack. ChIP-purified H-NS-DNA complexes were incubated with hydroxyl radicals for 0–4 min, and then the DNA was purified. Two segments (1 and 2) of the *LEE1* promoter region were detected by semi-quantitative PCR. As a control, from the same samples, DNA segment of *gadE* promoter region (P*gadE*) were detected by PCR. C. Effect of H-NS/StpA on the binding of PchA to the *LEE1* promoter region. ChIP-purified PchA-bound DNA from the W3110 wild type or the *hns stpA* mutant harboring pLux-P*LEE1* and pTB101-*pchA*-Strep were used as PCR templates for various segments (1 to 4).

A continuous, 600-bp, H-NS-bound DNA fragment corresponding to the promoter region of the *LEE1* operon was isolated from EHEC deficient in Pch expression (Fig 6A and S7 Fig). In the EHEC strain expressing PchA, the amount of the 600-bp DNA fragment was drastically reduced, as expected. Next, we compared the amounts of four segments, 150 bp each, which comprised the 600-bp *LEE1* promoter region. The relative amount of the DNA fragment covering the 5’ region of the *ler* gene in EHEC expressing PchA was low, as shown for the 600-bp sequence (Fig 6A segment 4). However, the binding of H-NS to the DNA fragment corresponding to the region upstream of the promoter was only slightly affected by the presence of PchA (Fig 6A segment 2).

To elucidate the conformational alterations, complexes containing H-NS and DNA were treated with hydroxyl radicals prior to the isolation of H-NS-bound DNA, and then specific sequences were detected by PCR. The sensitivity of the sequence in the 5’ part of the *ler* gene was affected by the expression of PchA; DNA fragments isolated from EHEC expressing PchA were more sensitive to hydroxyl radicals than those from EHEC deficient in the *pch* gene (Fig 6B segment 2). In contrast, the sensitivity of the upstream sequence was not affected by the expression of *pchA* (Fig 6A segment 1). These results clearly indicated that the conformation of the DNA-H-NS complex was altered by Pch expression, such that it became more sensitive to attack by hydroxyl radicals. Moreover, the H-NS bindings to the *gadE* promoter region, which expression was not activated by Pch [14], was unaffected by the presence of PchA, indicating that the reduction of H-NS binding by PchA is not caused by direct inhibition of binding ability of H-NS.

To explore the effects of H-NS and StpA on the binding of Pch, we also examined the binding of PchA at the *LEE1* promoter region in the presence or absence of H-NS/StpA. PchA bound four 150-bp segments of 600-bp regions, as examined for H-NS binding, and the amount of DNA bound to PchA was not affected by the presence of H-NS/StpA (Fig 6C). This finding suggested that the binding of PchA to the *LEE1* promoter region was unaffected by the presence of H-NS/StpA.

**Fig 7:**
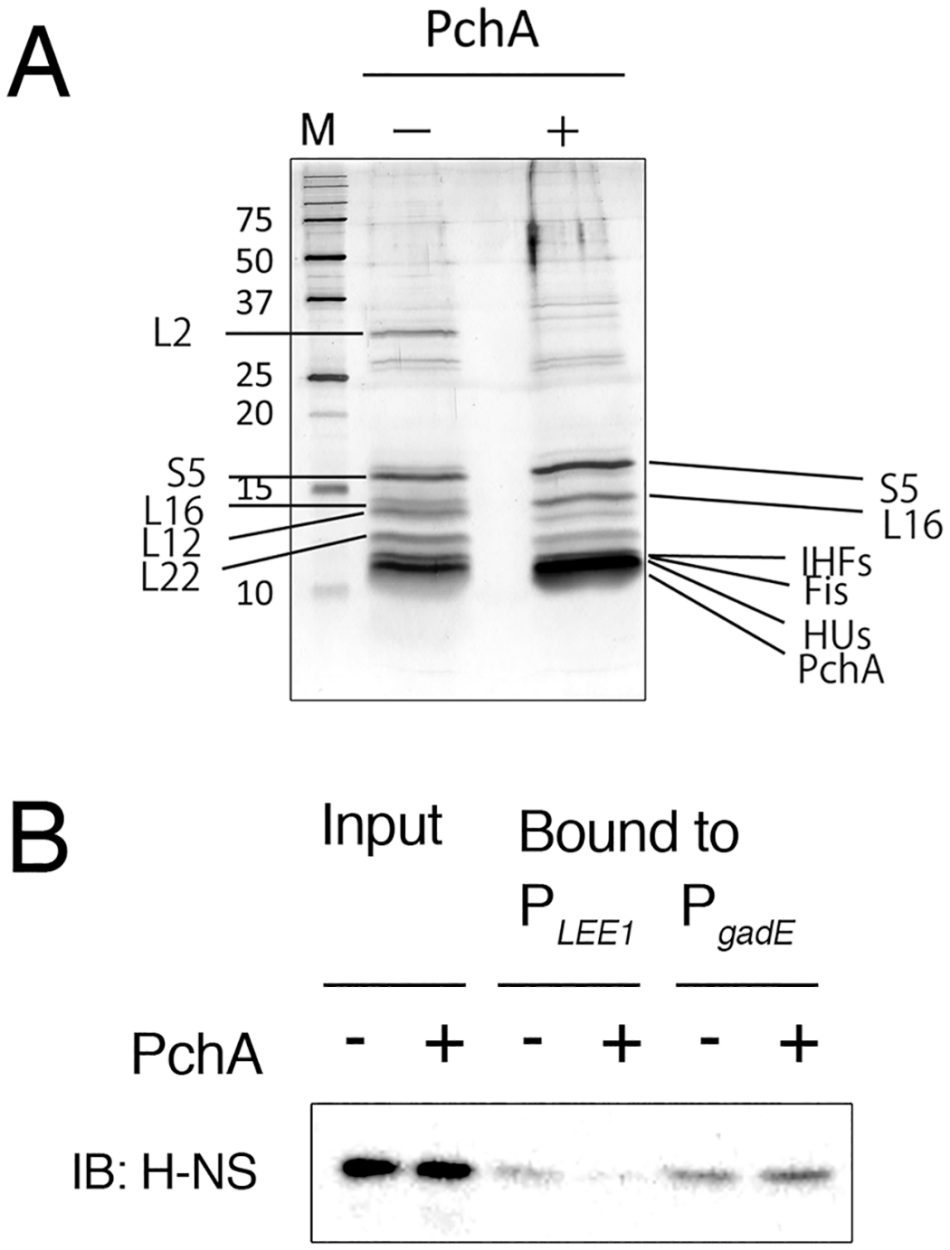
Reconstruction of the nucleoprotein complex on the *LEE1* promoter. Protein crude extract was prepared from W3110 harboring pTB101 (-pch) or from pTB101-*pch*-FLAG (+pch) and was incubated with a DNA fragment of the *LEE1* promoter immobilized on magnetic beads. A. Bound proteins were separated by SDS-PAGE and were visualized by silver staining, and major proteins were identified by LC-MS/MS. B. H-NS in the DNA-bound samples. H-NS in samples of the *LEE1* promoter DNA (P_*LEE1*_)-bound proteins (Bound) and crude protein extract (Input) were examined by immunoblotting using anti-H-NS antiserum. As a control, *gadE* promoter DNA (P_*gadE*_) was used to isolate promoter bound proteins from the same extracts.

To further examine the exclusive effect of Pch on H-NS binding, reconstruction of the H-NS-containing nucleoprotein complex was performed with protein crude extract of W3110 harboring pTB101-*pchA* or pTB101 (vector control). After incubation of immobilized *LEE1* promoter DNA on magnetic beads with crude protein extract, the beads were isolated and washed. Proteins bound to immobilized *LEE1* promoter DNA were extracted and separated by gel electrophoresis, and major proteins were then identified by LC-MS/MS. Although many of major proteins observed in gel were ribosome subunit proteins, several types of nucleoid proteins, such as IHF, HU, and Fis, were found in both preparations (Fig 7A). To confirm the difference in content of H-NS, the samples were subjected to immunoblotting using an H-NS-specific antibody. The results clearly showed that the amount of H-NS was much lower in the preparation from the Pch-containing extract than in the preparation from the Pch-negative extract (Fig 7B). In contrast, the same amount of H-NS was detected when *gadE* promoter DNA was used (Fig 7B). This result supported the inhibitory effect on H-NS binding of Pch protein.

## Discussion

**Fig 8:**
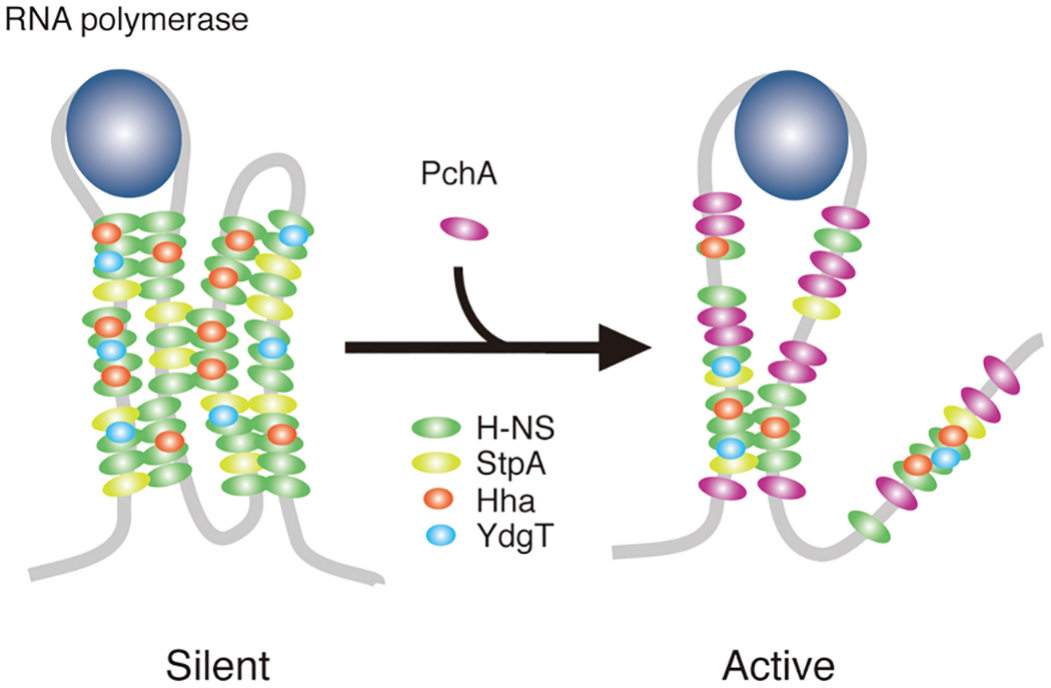
A schematic model of the Pch-mediated activation of the *LEE1* promoter. The nucleoid complex is composed of H-NS, StpA, Hha and YdgT. The role of YdgT is uncertain but it could be a member of the complex. In the silencing complex, DNA is folded through bridging between the proteins, and RNA polymerase might be trapped (silent). Competitive binding of Pch removes some H-NS and other proteins from the complex or inhibits the binding of them, resulting in the relaxed complex form, in which RNA polymerase can start transcription (active).

Most of virulence genes in EHEC are horizontally transferred genes, and their expression is repressed by H-NS when grown under normal conditions. Once they reach the site for infection, the expression is believed to be activated in response to changes in the environment. EHEC possess global regulators, Pch and Ler, which can activate a set of genes mostly associated with LEE-encoded T3SS and its secreted virulence proteins and effectors [14]. Although it has been shown that Ler is a homolog of H-NS and binds competitively to H-NS-binding regions, the activation mechanism for Pch has remained unknown. In this study, we showed that Pch activated target promoters through the disruption of the nucleoprotein complex formed with H-NS and related proteins, leading to de-repression. It was supported by the observation that, long DNA segment in H-NS-containing complex including the *LEE1* promoter region is reduced in the presence of the *pch* gene more than shorter segments of up-and down-stream of the promoter, and amount of H-NS bound to *LEE1* promoter DNA was greatly reduced by the protein extract from *E*. *coli* possessing the *pch* gene in in vitro reconstruction of nucleoid complex. Since global analysis of H-NS bindings on *E*. *coli* chromosome has shown that RNA polymerase association to the H-NS bound promoter is a general mode of transcription repression[7], we propose one of possible mechanisms that the exclusion of H-NS and related proteins from some position of the complex by Pch binding triggers the reduction of repression activity and provided room for RNA polymerase to initiate transcription (Fig 8).

The virulence genes in the LEE and the T3SS effector gene were positively regulated by Pch and Ler, which bound to the DNA at or around the genes [14]. Although transcription of the LEE genes, except for the *LEE1* operon, is believed to be directly regulated by Ler and Pch is involved indirectly in this regulation through its activation of *ler* expression, we showed that the full expression of the LEE genes was only achieved by expressing both Ler and Pch, which was the result of the direct contribution of Pch to activation of promoters for LEE operons and genes. The H-NS-binding profile at the LEE region was slightly altered in the strain expressing only Ler. In contrast, the simultaneous expression of both Pch and Ler regulators drastically reduced the amount of H-NS-bound DNA at the LEE region. Because Pch without Ler barely activated the transcription of the LEE genes, except for the *LEE1* operon [14], Pch might somehow enhance Ler-mediated transcriptional activation by binding to the same region. It is unlikely that Pch interacts with Ler directly to support the Ler-DNA interaction because Pch does not bind to Ler (unpublished observation) and because Pch can bind to DNA without Ler at many chromosomal loci [14]. The binding of Pch at the LEE regions might affect the nucleoprotein complex structure and enhance the replacement of H-NS with Ler and Pch. These results indicated that the efficient reduction of H-NS binding at virulence loci and the activation of virulence genes required both Pch and Ler. Furthermore, Pch played dual roles in the positive regulation of virulence genes as a transcriptional activator and as a co-activator of Ler on Ler-dependent promoters.

By comparing the promoter activities in *E*. *coli* with a variety of combinations of mutations of *hns* and its related genes, including *stpA*, *hha*, and *ydgT*, we concluded that H-NS was the main factor in silencing the *LEE1* promoter and that the StpA and Hha proteins were additional minor factors contributing to repression to some extent. Because the binding profile of StpA at the *LEE1* promoter and other chromosomal parts is almost the same as that of H-NS, the DNA-binding property of StpA could be the same as H-NS and StpA could be a component of the nucleoprotein complex containing H-NS at the *LEE1* promoter. Hha also contributes to the repression of the *LEE1* promoter, indicating that the nucleoprotein complex for promoter silencing at the *LEE1* promoter consists of at least H-NS/StpA/Hha. Although contribution of YdgT on silencing complex formation at *LEE1* promoter is small, deletion of *ydgT* in addition to *hha* deletion mutation increased the *LEE1* promoter activity, suggesting that YdgT somewhat affects the *LEE1* transcription. One of possibilities is that nucleoprotein complex containing YdgT affect elongation or release from pausing after transcription initiation. In contrast to the *LEE1* promoter, Pch can only slightly activate the *LEE2* promoter without Ler. Because Pch enhanced the promoter activity when Ler was working, Pch could also affect the nucleoprotein complex at the *LEE2* promoter. The effects of *hha* and *ydgT* deletion were much more obvious on adherence capacity, which depends on LEE gene expression levels (S8 Fig). The results suggested that the nucleoprotein complex formed at the *LEE1* promoter region was different from that formed at other LEE promoters using Hha/YdgT, which requires Ler for de-silencing.

Our results also suggested that the binding of Pch inhibited the binding of H-NS at specific local sequences, thus blocking the formation of the H-NS-DNA complex and preventing gene silencing. In EPEC *LEE1* promoter, the binding of H-NS to the NSR-II site, which overlaps with promoter region, has shown to be necessary for repression [25]. Though our results indicated that H-NS binds larger area, binding of H-NS to NRS-II site could be a key to the formation of repressor complex. Our observations suggested that Pch-mediated reduction of H-NS binding resulted in the formation of a relaxed nucleoprotein complex that is active for transcription (S7 Fig). This finding was confirmed by the experiments for ChIP-PCR with shorter segments and the reconstruction of the nucleoprotein complex on *LEE1* promoter DNA. The DNA segment containing the *LEE1* promoter was more sensitive to hydroxyl radicals when Pch was present, and much less of an amount of H-NS could bind to *LEE1* promoter DNA when PchA was contained in the same protein mixture, compared with the result when a Pch-negative mixture was used. H-NS binding at the region containing the *LEE1* promoter, NRS-II site, could be the primary target of Pch. It is unlikely that removal of H-NS from its target sites is the result of inhibitory interaction of Pch with H-NS, since Pch-affected H-NS bindings were limited to virulence loci and our attempt to co-precipitate H-NS with PchA did not show any interaction between them (unpublished observation). From the analysis for the *LEE1* promoter of EPEC, It was suggested that PerC acts as a derepressor as well as a transcriptional activator. In the report the transcription of the *LEE1* promoter was shown to be enhanced by the presence of *perC* gene in the *hns* deletion mutant [25]. Our results showed that in the *hns* mutant the presence of the *pchA* gene also enhanced the *LEE1* promoter activity but additional deletion of the *stpA* gene completely abolished the positive effect of the *pchA* gene. The results indicated that positive effect observed in the *hns* mutant is the results of counteraction against repression by the remaining gene products in the mutant such as StpA and Hha. Therefore, the horizontally acquired factor Pch could modify the nucleoprotein complex formed with H-NS and its related proteins, resulting in the de-silencing of virulence genes.

To acquire new traits and to incorporate them into the virulence regulatory system, a strategy to counteract the host transcription-repression mechanism would be efficient because a strict upstream activation sequence for regulatory factors is not necessary. The global regulatory system consisting of Pch and Ler provides a flexible, easy-to-expand capability for bacteria to adapt to new environments or to evolve advanced infectious strategies.

## Materials and Methods

### Bacterial strains, plasmids, and culture conditions

EHEC O157 Sakai (RIMD 0509952) [17] and its derivative strains as well as the plasmids used in this study are listed in S1 Table. The deletion mutants and *hns*-FLAG derivatives of EHEC O157 Sakai were constructed using the methods and plasmids of Datsenko and Wanner [34]. Derivatives of W3110 mutants for *hns* and/or *stpA*, *hha*, and *ydgT* were constructed by P1 transduction, using the corresponding mutants in our stock as donors [30]. pWKS-P*tac-ler* was constructed by replacing the P*tac* promoter DNA with the upstream sequence of the *ler* gene on pWKS-*ler*. Bacteria were grown overnight in LB, diluted 100-fold, and then incubated in DMEM (Sigma) at 37°C with shaking to 0.9–1.0 OD_600_. To examine the effects of IPTG on the expression of LEE genes, bacteria were grown overnight in LB, diluted 100-fold, and then grown in DMEM containing 0–400 μM IPTG at 37°C with shaking to 0.9–1.0 OD_600_.

### Analysis of proteins in whole-cell lysates

Bacteria were collected from cultures by centrifugation, and the cell pellet was dissolved in SDS sample buffer. The concentration of each sample was normalized to the OD_600_ of the culture, and samples prepared from similar numbers of cells were analyzed by immunoblotting. The proteins were separated by SDS-polyacrylamide (12% or 10%) gel electrophoresis (SDS-PAGE) and were transferred onto an Immobilon membrane (Millipore). The proteins were detected with antibodies specific for EspB, Tir [35], or DnaK (Calbiochem) and a horseradish peroxidase-conjugated secondary antibody, followed by visualization with an ECL detection kit (Amersham Biosciences).

### Promoter activity assay

The P*LEE1*-*gfp* operon fusion plasmid pACYC-P*LEE1-gfp* or the P*LEE1*-*lux* operon fusion plasmid pLux-P*LEE1* [36] was used to measure the promoter activity of the *LEE1* operon. Briefly, bacteria were grown overnight in LB and were diluted 100-fold with DMEM containing various amounts of IPTG. For fluorescence, at the sampling time points, an aliquot of culture was removed, and the bacteria were collected by centrifugation, washed with PBS, and suspended in PBS. The OD_600_ was measured, and the intensity of the fluorescence was measured by an FP-6200 (Jasco). The fluorescence intensity was normalized to the OD_600_ value. The average of three independent experiments was presented. For luciferase activity, at the sampling time points, 800 μl was removed to measure the OD_600_, and 100 μl was obtained to measure the luminescence intensity, using a TD-20/20 luminometer (Turner Biosystems). The luciferase activity was calculated by dividing the luminescence intensity by the OD_600_. The average and standard error were calculated from the results of three experiments.

### Adherence assay

The adherence assay was performed as previously described [35], with a slight modification. Bacteria were grown overnight in LB, diluted 100-fold in DMEM with IPTG (0–400 μM), and incubated at 37°C for 3 h with shaking. Caco-2 cells in a confluent monolayer were infected at an m.o.i. (multiplicity of infection) of 100 +/- 15 for 2 h at 37°C. The cells were washed with PBS and then were incubated in fresh medium for an additional 3 h at 37°C. After another PBS wash, the cells were fixed and stained for bacteria with anti-O157 LPS and Alexa488-conjugated anti-rabbit IgG or for F-actin with rhodamine-phalloidin, as previously described [37].

### ChIP-on-chip

Chromatin immunoprecipitation and DNA microarray analysis were performed as described by Oshima et al. (2006) and by Abe et al., (2008) with slight modifications. Derivatives of the EHEC O157 Sakai strain were grown in DMEM with or without 6 μM IPTG to logarithmic phase (OD_600_ = 0.7–0.8) at 37°C. Formaldehyde (final concentration of 1%) was added to 10 ml of the culture, and the mixture was incubated at room temperature for 20 min. To terminate the crosslinking reaction, glycine (final concentration of 0.45 M) was added, and the mixture was incubated at room temperature for 5 min. Bacteria were collected by centrifugation and were washed first with TBS and then twice with washing buffer (50 mM Na phosphate [pH 8.0], 300 mM NaCl, 0.01% Tween-20), were suspended in 1 ml of lysis buffer (50 mM Na phosphate [pH 8.0], 300 mM NaCl, 1% Tween-20, 20 mg/ml lysozyme) and were stored at -20°C until use.

For analysis, the bacteria were incubated at 37°C for 30 min, diluted in 4 ml of washing buffer containing PMSF (final concentration of 1 mg/ml), and then lysed by sonication using a UCD-200 Bioruptor (Cosmo Bio Co., Ltd.) at 4°C with 15 30-sec pulses at 30-sec intervals. The cell debris was removed by centrifugation at 15,000 rpm for 30 min. An 800-μl aliquot of the supernatant was mixed with Protein G-conjugated magnetic beads (Dynabeads) bound to anti-FLAG (M2 monoclonal antibody; Sigma-Aldrich) and incubated at 4°C overnight. The beads were washed three times with washing buffer and then with TE. The protein-DNA complex bound to the beads was released into 100 μl of elution buffer (50 mM Tris-HCl [pH 7.5], 10 mM EDTA, 1% SDS) by heating at 65°C for 20 min. The DNA fragments that were cross-linked to proteins were released by incubating the eluate with proteinase K (1 mg/ml) at 42°C for 2 h and then at 65°C for 6 h, followed by purification using a Qiagen PCR Clean-up kit (Qiagen) in 200 μl of TE. The recovered DNA fragments were amplified, terminally labeled, and hybridized with the EHEC O157-tiled microarray, as previously described [7].

The raw intensity data (.CEL files) were processed using In Silico Molecular Cloning Array Edition software (In Silico Biology) for analysis and visualization. The signal intensities were obtained by subtracting the signal intensities of the mismatch probes from those of the perfect-match probes, and probes with negative values were excluded from further analysis. To compensate for differences among the probes caused by different probabilities of DNA disruption and hybridization efficiency, the signals were normalized by dividing them by the signal intensities from the DNA samples obtained before the beads were added. The binding sites were identified with In Silico Molecular Cloning Array Edition software (In Silico Biology) by selecting a region with continuous 10 or more signal ratios (ChIP signal/input signal) greater than the threshold (1.0). The resolution of the experiments are around 50 bp, because average density of DNA probe is 50bp and DNA fragments used for the experiments are ~200bp after sonication. The reproducibility and specificity of the binding of H-NS to several sites were confirmed by ChIP-PCR. The raw data (.CEL format) from the ChIP-chip experiments were deposited in the ArrayExpress database (http://www.ebi.ac.uk/microarray-as/ae/) under accession numbers E-TABM-819 and E-TABM-818.

### Hydroxyl radical treatment of the nucleoprotein complex

The H-NS-DNA complex, prepared as described above, was treated with hydroxyl radicals as follows, and then the DNA was decrosslinked and purified as described above. Fifty microliters of the samples was placed at the bottom of Eppendorf tubes, and 2.5 μl of Fe(II) EDTA solution (mixture of equal volume of 0.4 mM (NH_4_)_2_Fe(SO_4_)_2_ and 0.8 mM EDTA) and 2.5 μl of 20 mM sodium ascorbate were placed on the side wall of the tube. Treatment was started by short centrifugation and incubation for 1, 2 or 4 min at room temperature until the addition of 5.5 μl of 0.1 M thiourea and 0.5 μl of 0.2 M EDTA to stop the reaction.

### In vitro reconstruction of the nucleoprotein complex

Immobilized *LEE1* promoter DNA on magnetic beads was constructed by the binding of *LEE1* promoter DNA, which was isolated by PCR with biotin-labeled primers, to streptavidin-coated magnetic beads (Dynabeads M-280 Streptavidin; Invitrogen). Crude protein extract was prepared from W3110 harboring pTB101 or pTB101-*pchA*, and binding to immobilized DNA was performed as previously described [38]. The proteins bound to DNA were released in 40 ml of releasing buffer (10 mM Tris-Cl [pH 7.9], 1 mM EDTA, 5% glycerol, 1 mM DTT, 2.0 M NaCl). Proteins were separated by SDS-PAGE and visualized by silver stain, and major bands were subjected to LC-MS/MS for identification as previously described [37].

### Statistical analysis and experiment reproducibility

The statistical analysis was calculated using the build-in mathematical function of Excel (Microsoft). Difference between two groups was identified using the Student’s *t*-test under the condition of two-tails/two-sample unequal for all analysis. Data are presented as means with standard error of mean (S.E.M). All experiments were repeated for reproducibility and the representative data was shown in figures.

## Supporting Information

S1 Fig Growth of EHEC O157 harboring pTB101-pchA in LB containing various concentration of IPTG. After dilution of overnight culture 100-fold with LB containing various amount of IPTG (0, 1.5, 5, 25, 100 **μ**M), growth were monitored by measuring OD600.
(PDF)

S2 Fig Promoter activities in W3110 and its derivatives were measured at early stationary growth phase. A. Representative of cell density (OD_600_) at sampling for promoter assay. Promoter activity shown in Fig 3 was measured when cell density reached around 2.0 OD_600_. For example, wild type was measured at 4h, and the hns stpA double mutant was measured at 6h. B. Growth of wild type and mutants of W3110. After dilution of overnight culture 100-fold with LB, growth were monitored by measuring OD600. W3110 (square), *hns stpA* double mutant (circle), *hns stpA hha ydgT* quadruple mutant (triangle)
(PDF)

S3 Fig Distribution of H-NS binding in regions containing genes in the Pch/Ler regulon class L1. The H-NS, Pch, and Ler binding for four representative Pch/Ler-regulon gene loci is shown. The rows are the same as in Fig 2. The ORFs in orange are genes that belong to the Pch/Ler regulon class L1 (Abe et al., 2008). The ORFs in yellow are genes that belong to the Pch/Ler regulon class L2 (Abe et al., 2008).
(PDF)

S4 Fig Distribution of H-NS binding in the regions containing genes in the Pch/Ler regulon class L2. The H-NS, Pch, and Ler bindings for five representative Pch/Ler-regulon gene loci are shown. The rows are the same as in Fig 2. The ORFs in yellow are genes that belong to the Pch/Ler regulon class L2.
(PDF)

S5 Fig Comparison of the G+C content and size of H-NS binding sites between altered and unaltered H-NS binding by Pch/Ler expression. A. G+C content of H-NS binding sites. The effect of the expression of Pch and Ler on H-NS occupancy was determined by comparing H-NS binding profiles taken from the *pch* mutant, which does not express either *pch* or *ler*, and the *pch*-expression strain, which expresses both *pch* and *ler*. Affected: the H-NS binding sites with decreased occupancy in the *pch* expressing strain. B. Size distributions of H-NS binding sites.
(PDF)

S6 Fig Distribution of H-NS binding over the EHEC O157 Sakai chromosome and LEE region. A. H-NS binding profiles on the whole EHEC chromosome. Graphs on the three outer circles show H-NS binding to the chromosome of the EHEC Sakai strain. Graphs on the fourth and fifth circles represent Ler binding and PchA binding from previous data (Abe et al., 2008). The radial blue bars indicate the relative value of occupancy. The sixth circle shows the G+C content. The seventh circle shows the EHEC chromosome, including the positions and prophages or prophage-like elements. B. H-NS binding profiles in the LEE of EHEC O157 Sakai and its surrounding region. The top row shows the G+C content. The next three rows show the H-NS binding of EHEC deficient in *pch* and *ler* expression (Row 2), EHEC expressing Ler but not Pch (Row 3), and EHEC expressing both Pch and Ler (Row 4). Rows 5 and 6 show Ler and Pch binding, respectively. The bottom row shows the ORFs in the *E*. *coli* K12 common chromosome (blue) and the ORFs in the LEE and other laterally transferred elements (red).
(PDF)

S7 Fig Specificity of ChIP of H-NS-bound DNA fragments. After crosslinking the H-NS-DNA complexes were precipitated with Dynabeads TALON. DNA segments corresponding to *LEE1* promoter were detected by PCR. For control, uncharged Dynabeads protein G was used for precipitation. Samples were as input (i), precipitates with control Dynabeads (-), and precipitates with Dynabeads TALON (+).
(PDF)

S8 Fig Adherence of an EHEC strain possessing *hha* and/or *ydgT* mutation(s) to Caco-2 cells. Caco-2 cells were infected with wild-type EHEC, the *hha* mutant, the *ydgT* mutant or the *hha ydgT* mutant for 90 min. Unattached bacteria were then removed, and the cells were incubated for another 3 h. Microcolonies were visualized by Giemsa staining (A). The frequency of appearance of microcolonies is presented as the number of microcolonies per cell, calculated from a total of 5 microscopic sights and the average of three independent experiments for each strain (B).
(PDF)

S1 Table Strains and plasmids used in this study. (PDF)
