# Supplementary figures and images for: Gene Activation through the Modulation of Nucleoid Structures by a Horizontally Transferred Regulator, Pch, in Enterohemorrhagic Escherichia coli
Source: PLoS One. 2016 Feb 22;11(2):e0149718. doi: 10.1371/journal.pone.0149718 (PMC4764244; doi:10.1371/journal.pone.0149718)

S1 Fig

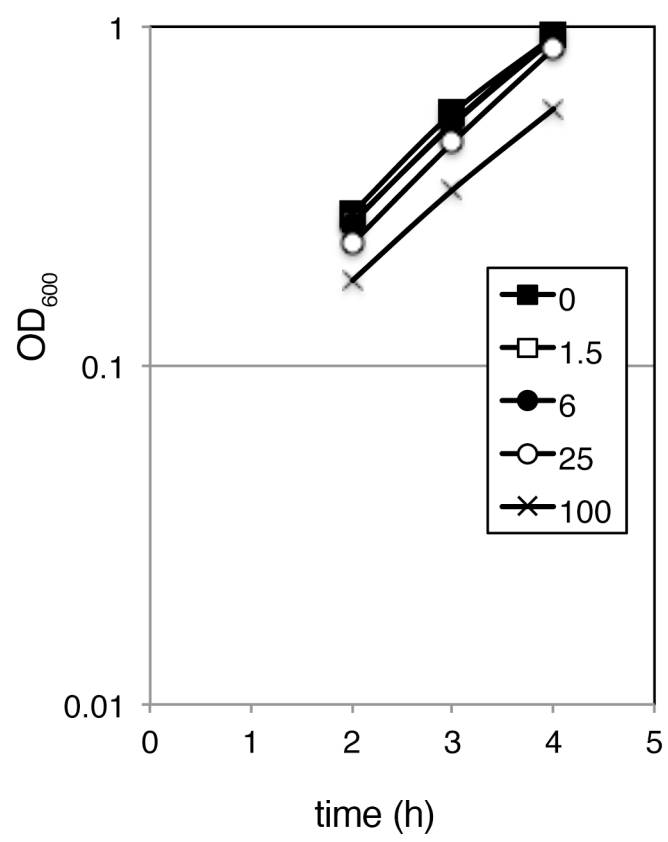

Supplement: S1 Fig — After dilution of overnight culture 100-fold with LB containing various amount of IPTG (0, 1.5, 5, 25, 100 μM), growth were monitored by measuring OD600. (PDF) [file pone.0149718.s001.pdf]

S3 Fig

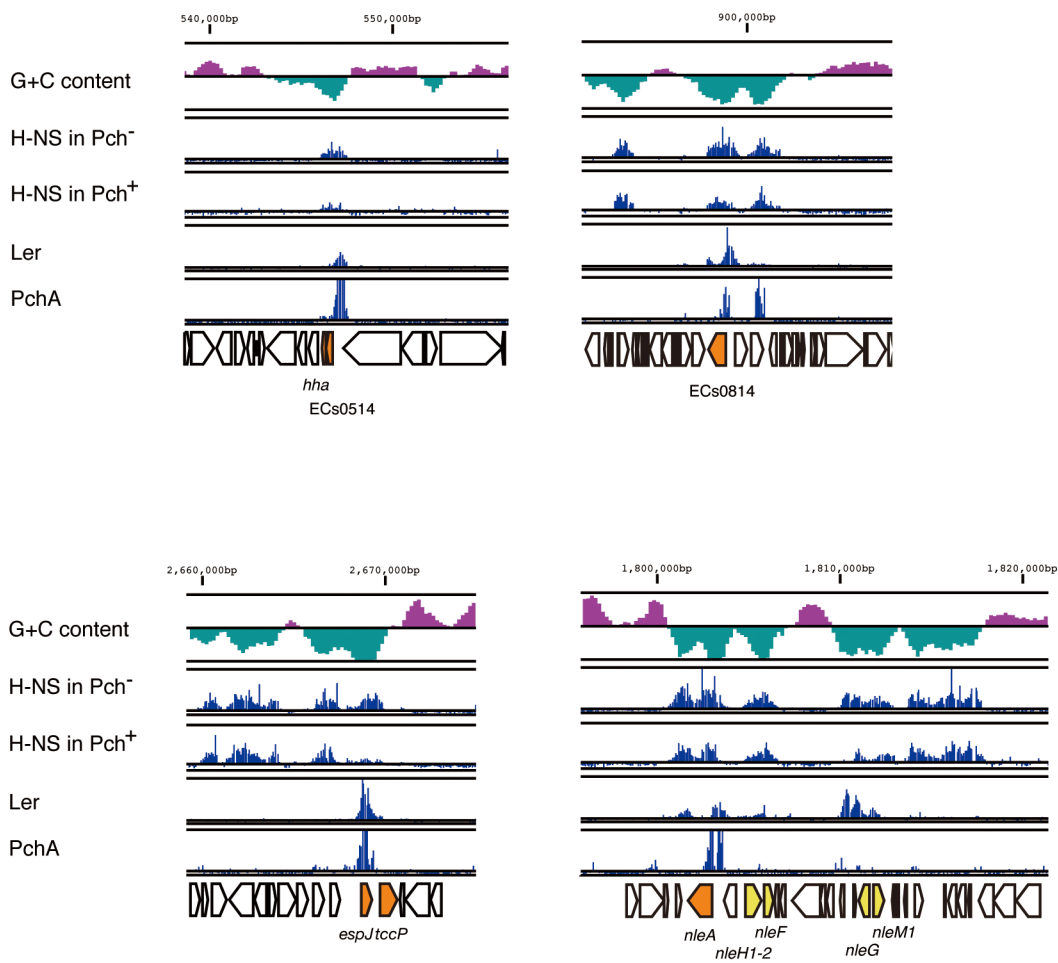

Supplement: S3 Fig — The H-NS, Pch, and Ler binding for four representative Pch/Ler-regulon gene loci is shown. The rows are the same as in Fig 2. The ORFs in orange are genes that belong to the Pch/Ler regulon class L1 (Abe et al., 2008). The ORFs in yellow are genes that belong to the Pch/Ler regulon class L2 (Abe et al., 2008). (PDF) [file pone.0149718.s003.pdf]

S4 Fig

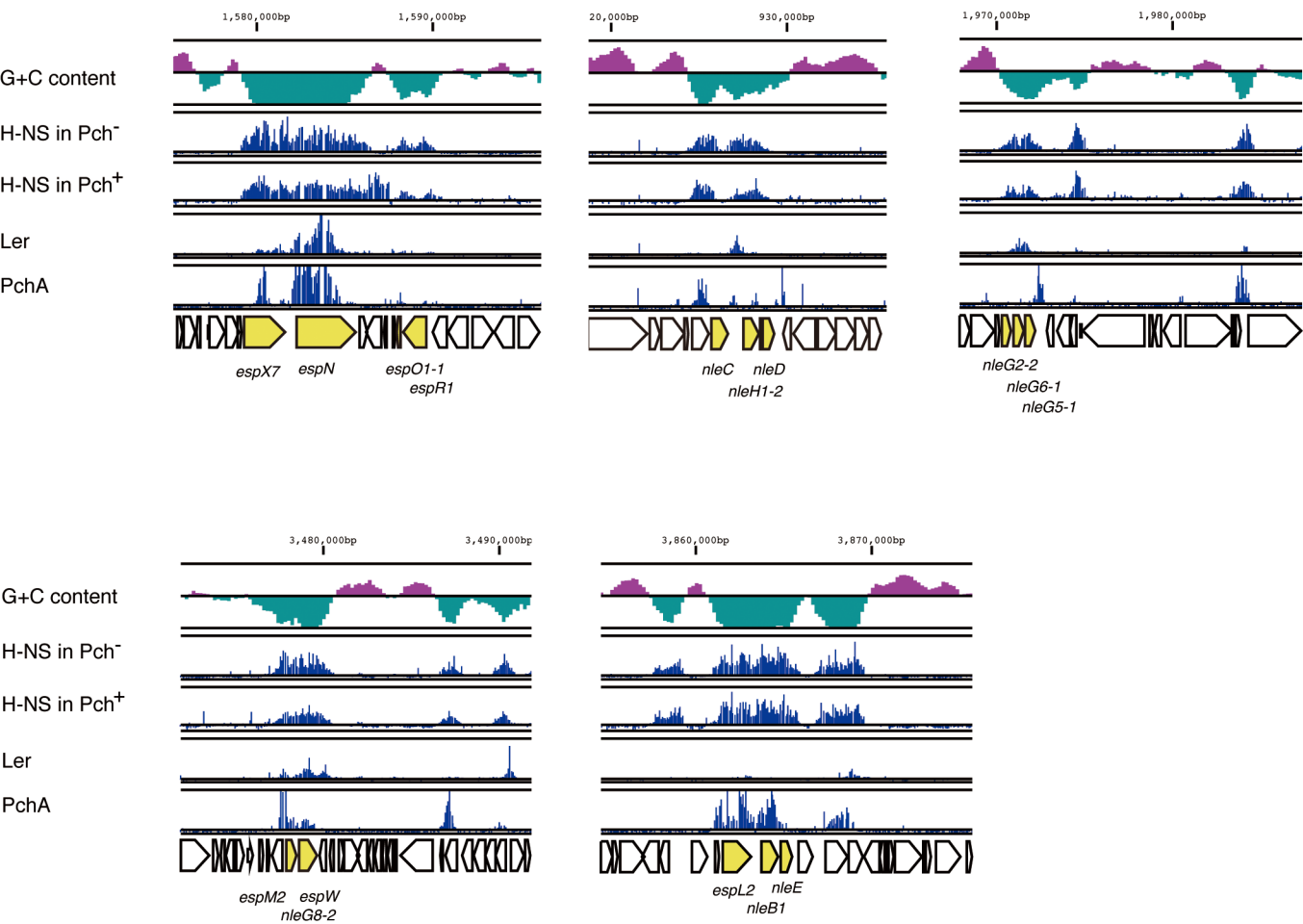

Supplement: S4 Fig — The H-NS, Pch, and Ler bindings for five representative Pch/Ler-regulon gene loci are shown. The rows are the same as in Fig 2. The ORFs in yellow are genes that belong to the Pch/Ler regulon class L2. (PDF) [file pone.0149718.s004.pdf]

S5 Fig

**A**

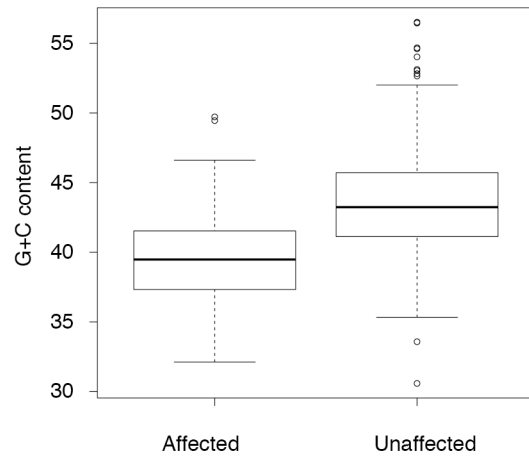

**B**

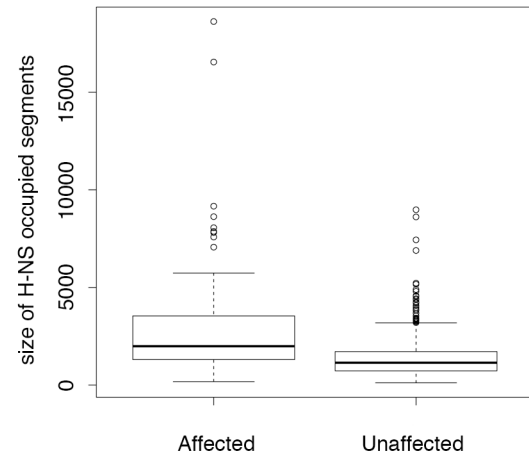

Supplement: S5 Fig — A. G+C content of H-NS binding sites. The effect of the expression of Pch and Ler on H-NS occupancy was determined by comparing H-NS binding profiles taken from the pch mutant, which does not express either pch or ler, and the pch-expression strain, which expresses both pch and ler. Affected: the H-NS binding sites with decreased occupancy in the pch expressing strain. B. Size distributions of H-NS binding sites. (PDF) [file pone.0149718.s005.pdf]

S6 Fig

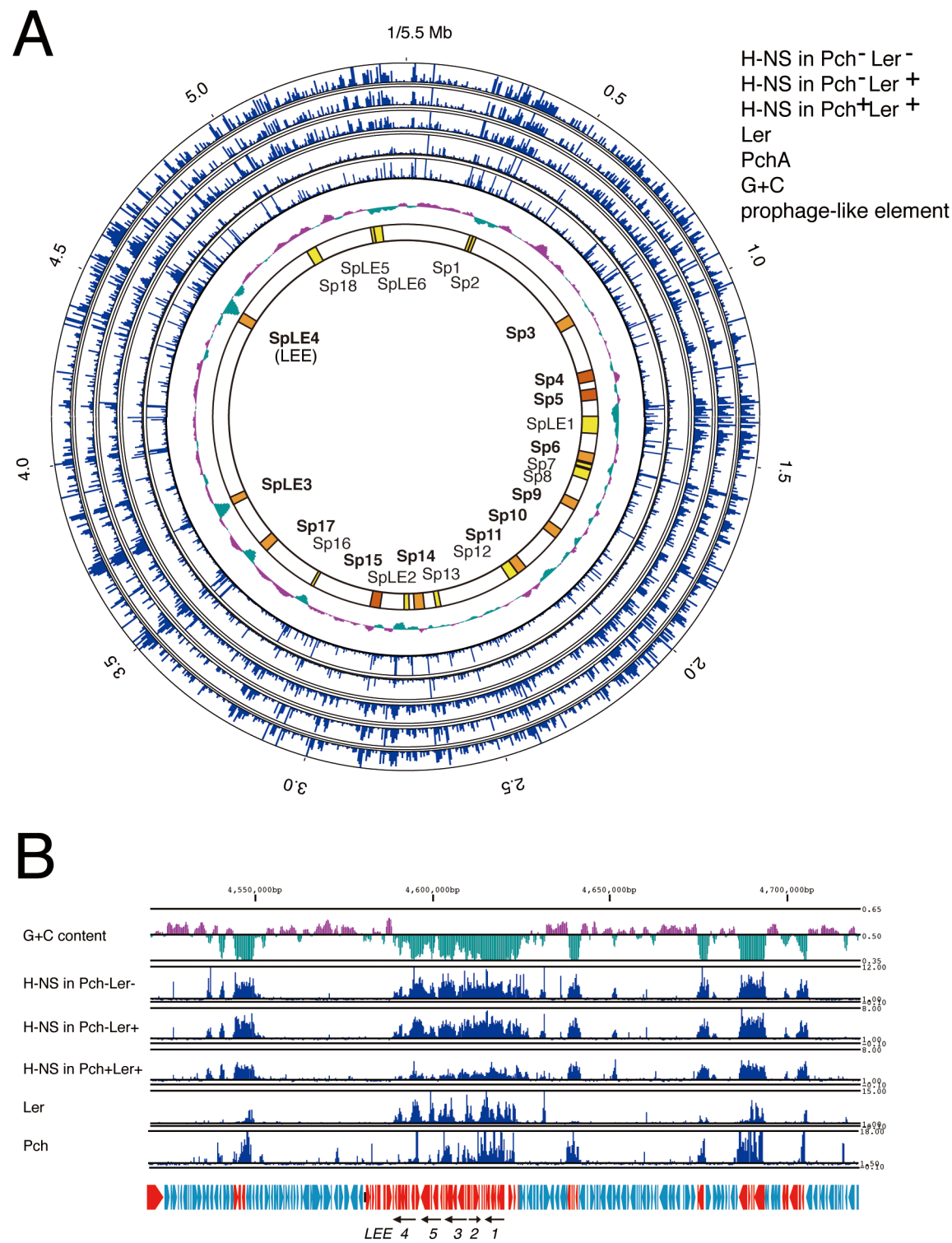

Supplement: S6 Fig — A. H-NS binding profiles on the whole EHEC chromosome. Graphs on the three outer circles show H-NS binding to the chromosome of the EHEC Sakai strain. Graphs on the fourth and fifth circles represent Ler binding and PchA binding from previous data (Abe et al., 2008). The radial blue bars indicate the relative value of occupancy. The sixth circle shows the G+C content. The seventh circle shows the EHEC chromosome, including the positions and prophages or prophage-like elements. B. H-NS binding profiles in the LEE of EHEC O157 Sakai and its surrounding region. The top row shows the G+C content. The next three rows show the H-NS binding of EHEC deficient in pch and ler expression (Row 2), EHEC expressing Ler but not Pch (Row 3), and EHEC expressing both Pch and Ler (Row 4). Rows 5 and 6 show Ler and Pch binding, respectively. The bottom row shows the ORFs in the E. coli K12 common chromosome (blue) and the ORFs in the LEE and other laterally transferred elements (red). (PDF) [file pone.0149718.s006.pdf]

S7 Fig

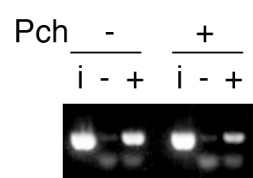

Supplement: S7 Fig — After crosslinking the H-NS-DNA complexes were precipitated with Dynabeads TALON. DNA segments corresponding to LEE1 promoter were detected by PCR. For control, uncharged Dynabeads protein G was used for precipitation. Samples were as input (i), precipitates with control Dynabeads (-), and precipitates with Dynabeads TALON (+). (PDF) [file pone.0149718.s007.pdf]

S8 Fig

A

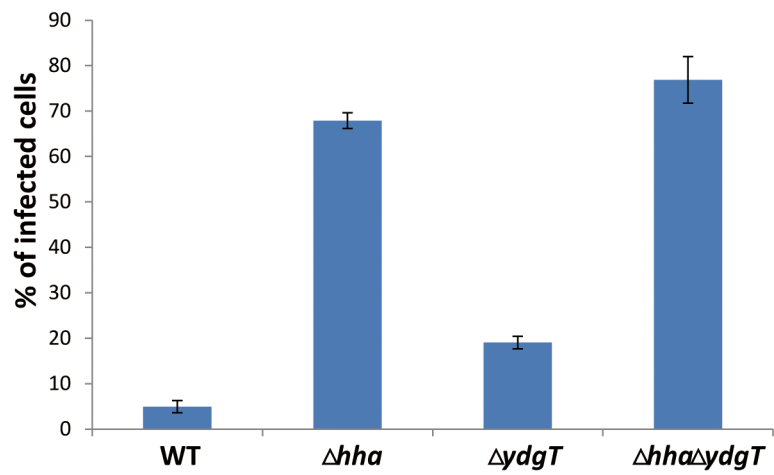

B

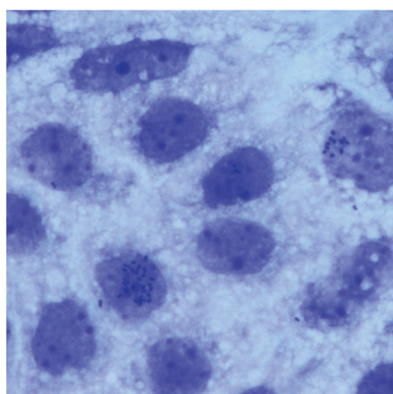

WT

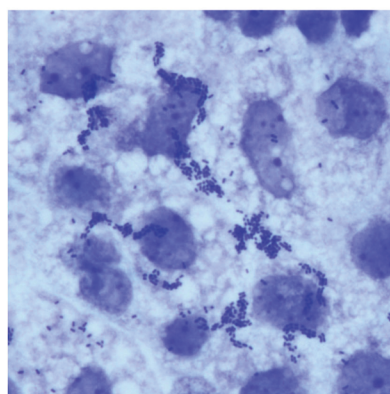

$\Delta hha$

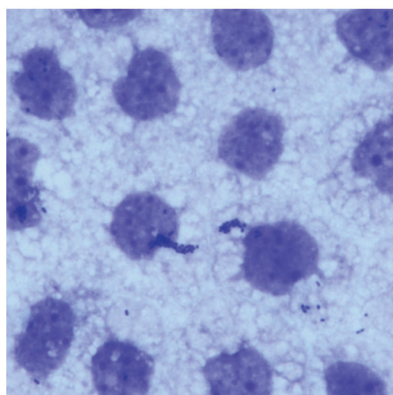

$\Delta ydgT$

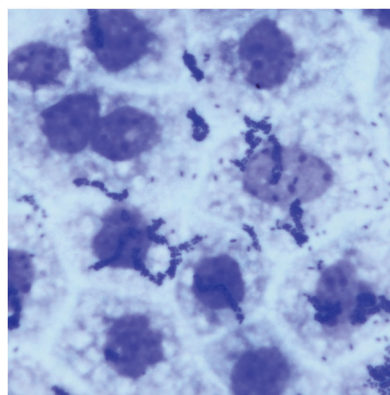

$\Delta hha\Delta ydgT$

Supplement: S8 Fig — Caco-2 cells were infected with wild-type EHEC, the hha mutant, the ydgT mutant or the hha ydgT mutant for 90 min. Unattached bacteria were then removed, and the cells were incubated for another 3 h. Microcolonies were visualized by Giemsa staining (A). The frequency of appearance of microcolonies is presented as the number of microcolonies per cell, calculated from a total of 5 microscopic sights and the average of three independent experiments for each strain (B). (PDF) [file pone.0149718.s008.pdf]
