# Supplementary material for: Gene Activation through the Modulation of Nucleoid Structures by a Horizontally Transferred Regulator, Pch, in Enterohemorrhagic Escherichia coli
Source: PLoS One. 2016 Feb 22;11(2):e0149718. doi: 10.1371/journal.pone.0149718 (PMC4764244; doi:10.1371/journal.pone.0149718)

S2 Fig.

A

OD600	pch−	pch+
WT	2.090	2.127
⊿hns	2.026	2.031
⊿stpA	2.012	2.102
⊿ydgT	2.031	2.133
⊿hha	1.961	1.957
⊿ydgT⊿stpA	1.990	2.101
⊿hha⊿stpA	1.920	1.911
⊿hha⊿ydgT	1.870	1.926
⊿hns⊿ydgt	1.938	1.928
⊿hns⊿hha	1.850	1.841
⊿hns⊿stpA	2.199	1.979
⊿hns⊿hha⊿ydgT	1.960	1.854
⊿hns⊿stpA⊿ydgT	1.802	1.798
⊿hns⊿stpA⊿hha	1.824	1.947
⊿hns⊿stpA⊿hha⊿ydgT	1.896	1.906

B

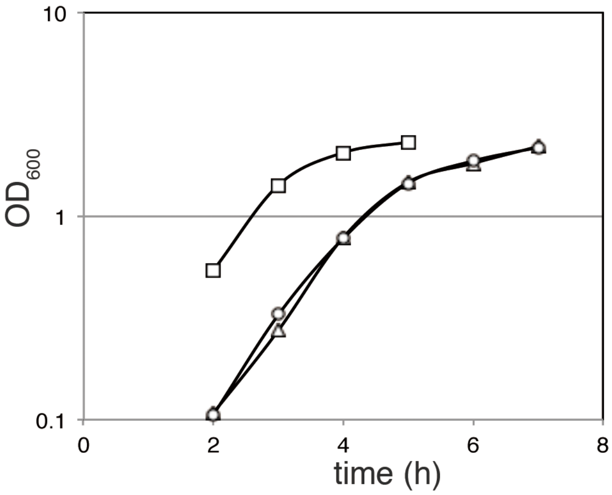

Supplement: S2 Fig — A. Representative of cell density (OD600) at sampling for promoter assay. Promoter activity shown in Fig 3 was measured when cell density reached around 2.0 OD600. For example, wild type was measured at 4h, and the hns stpA double mutant was measured at 6h. B. Growth of wild type and mutants of W3110. After dilution of overnight culture 100-fold with LB, growth were monitored by measuring OD600. W3110 (square), hns stpA double mutant (circle), hns stpA hha ydgT quadruple mutant (triangle) (PDF) [file pone.0149718.s002.pdf]
