# Supplementary material for: Gene Activation through the Modulation of Nucleoid Structures by a Horizontally Transferred Regulator, Pch, in Enterohemorrhagic Escherichia coli
Source: PLoS One. 2016 Feb 22;11(2):e0149718. doi: 10.1371/journal.pone.0149718 (PMC4764244; doi:10.1371/journal.pone.0149718)
Supplement: S1 Table — (PDF) [file pone.0149718.s009.pdf]

S1 Table. Strains and plasmids

Strain or Plasmid	Description	Reference
Strain		
Sakai (RIMD 0509952)	Wild type EHEC O157:H7	Hayashi *et al*., 2001
SKI 1182	Sakai Δ*pchA*Δ*pchB*Δ*pchC*	Abe et al., 2009
SKI 1258	Sakai *hns*-FLAG	This study
SKI 1525	Sakai *stpA*-FLAG	This study
SKI 1394	Sakai Δ*pchA*Δ*pchB*Δ*pchC hns*-FLAG	This study
SKI 1612	Sakai Δ*pchA*Δ*pchB*Δ*pchC stpA*-FLAG	This study
SKI 1271	Sakai Δ*ler hns*-FLAG	This study
SKI 1282	Sakai Δ*pchB*Δ*pchC pchA*-Strep	Abe et al., 2009
SKI 1172	Sakai *ler*-FLAG	Abe et al., 2009
W3110	Wild type *E. coli* K-12	lab stock
W3110 *hns*	W3110 Δ*hns*	This study
W3110 *stpA*	W3110 ΔstpA	This study
W3110 *ydgT*	W3110 ΔydgT	This study
W3110 *hha*	W3110 Δ*hha*	This study
W3110 *stpA ydgT*	W3110 Δ*stpA* Δ*ydgT*	This study
W3110 *stpA hha*	W3110 Δ*stpA* Δ*hha*	This study
W3110 *ydgT hha*	W3110 Δ*ydgT* Δ*hha*	This study
W3110 *hns ydgT*	W3110 Δ*hns* Δ*ydgT*	This study
W3110 *hns hha*	W3110 Δ*hns* Δ*hha*	This study
W3110 *hns stpA*	W3110 Δ*hns* Δ*stpA*	This study
W3110 *hns ydgT hha*	W3110 Δ*hns* Δ*ydgT* Δ*hha*	This study
W3110 *hns stpA ydgT*	W3110 Δ*hns* Δ*stpA* Δ*ydgT*	This study
W3110 *hns stpA hha*	W3110 Δ*hns* Δ*stpA* Δ*hha*	This study
W3110 *hns stpA ydgT hha*	W3110 Δ*hns* Δ*stpA* Δ*ydgT* Δ*hha*	This study
Plasmid		
pTB101-*pchA*	pTB101 carrying Ptac-pchA fusion	Abe et al., 2009
pTB101-*ler*	pTB101 carrying Plac-ler fusion	Abe et al., 2009
pSU-*pchA*	Plac-pchA fusion	This study
pSU-gfp-P*LEE1*	PLEE1-gfp operon fusion	This study
pLux-P*LEE1*	P*LEE1*-luciferase operon fusion	Takao *et al*., 2014
pLux-P*LEE2*	P*LEE2*-luciferase operon fusion	This study

Abe, H. et al. DNA Res 15, 25-38 (2008).
Hayashi, T. et al. DNA Res 8, 11-22 (2001).
Nakanishi, N. et al. Microbiology 155, 521-30 (2009).
Takao, M. et al. Mol Microbiol 93, 1302-1313 (2014).
